# Editorial: The contribution of molecular priming to abiotic stress tolerance in plants

**DOI:** 10.3389/fpls.2023.1352312

**Published:** 2024-01-12

**Authors:** Ece Turhan, Mohd Asgher

**Affiliations:** ^1^ Department of Agricultural Biotechnology, Faculty of Agriculture, Eskisehir Osmangazi University, Eskisehir, Türkiye; ^2^ Plant Physiology and Biochemistry Laboratory, Department of Botany, Baba Ghulam Shah Badshah University, Rajouri, Jammu and Kashmir, India

**Keywords:** abiotic stress, biostimulants, exogenous applications, gene expression, molecular priming, signalling molecules

Extreme climate change such as drought, temperature changes, flooding, and heavy metals affecting important biological processes like gene expression, cell metabolism, growth and development ultimately reduce crop production worldwide (Zhang et al., 2023). To meet increasing demands for food, it is critical to reduce these losses (Razzaq et al., 2021). Several strategies, including molecular priming, have been developed in recent years to lessen the detrimental impacts of abiotic stressors on agricultural production, in addition to traditional breeding and biotechnology procedures. Abiotic stressors are responsible for a reduction in crop productivity through the generation of reactive oxygen species (ROS) (Asgher et al., 2023). To cope with and battle severe environmental stress, plants have evolved intricate defence systems. Several exogenous treatments have been shown to increase the natural defences in plants by improving growth and some critical physiological processes such as mineral uptake and antioxidant enzyme activity, resulting in stress reduction (Turhan et al., 2020).

This Research Topic includes contributions by invited investigators, with original research articles as well as review articles that aim to understand the contributions of molecular priming to abiotic stress tolerance in plants. The Research Topic includes four original research papers and a review, all of which aim to eliminate the negative effects of abiotic stress in plants using the molecular priming method and to comprehend the use of this methodology at the molecular level.

Recent evidence from Talaat (2023) has shown that the exogenous application of signalling molecules such as melatonin improves the water stress tolerance of the *Hordeum vulgare* by reprogramming endogenous plant hormone production and antioxidant enzyme activities, which in turn promotes membrane stability and photosynthesis. Furthermore, recent research has provided that a number of signalling molecules are employed in abiotic stress tolerance such as phytohormones and non-proteogenic amino acids such as N-acetylglutamic acid (NAG). Molecular priming is an environmentally friendly technology to counteract the ill effects of abiotic stresses on a diverse range of crop plants (Kerchev et al., 2020). Crops treated with the fungus Trichoderma have the potential to control disease and pests and also alleviate abiotic stresses (Brotman et al., 2013). Arbuscular mycorrhiza, as a natural root symbiont, provides essential plant inorganic nutrients to host plants, thereby improving growth and yield and protecting plants against abiotic stress. In response to abiotic stress, plants have produced coping mechanisms that enable them to thrive in adverse environments (Zhang et al., 2023). Chemical priming is an important strategy for mitigating abiotic stress in crop plants; compounds used for this purpose have been shown to work in a wide range of plant species and increase tolerance to a variety of abiotic stresses (Sako et al., 2020).

The articles published in the Research Topic comprehensively explain the role of molecular priming, enabling crop plants to be safer against abiotic stress. Dong et al. studied how arbuscular mycorrhizal fungi increased the saline-alkali tolerance in *Populus simonii×P. nigra* by establishing a symbiotic relationship and improved photosynthesis and growth. Arbuscular mycorrhizal fungus may impart this enhanced tolerance by changing the expression of the NHX gene family. The plants were inoculated with *Funneliformis mosseae* improved chlorophyll fluorescence and photosynthetic attributes and encouraged the absorption of water, K^+^, and Ca^++^ under saline alkali stress. They also discovered eight NHX gene families in *Populus simonii*x*P. nigra* and uncovered that *F. mosseae* modulates Na^+^ distribution by stimulating the expression of PxNHXs. Besides, alterations in gene expression decreased pH and Na^+^ concentrations in rhizosphere soil while improving the soil environment.

Niu et al. address the role of *Trichoderma harzianum* in alleviating waterlogging injury in tomatoes. They found that two tomato genotypes (waterlogging tolerant; ‘MIX-002) and (waterlogging sensitive; ‘LA4440’) responded differently to waterlogging priming and stress in terms of photosynthesis, and ROS. Both the tomato genotypes exhibited distinct photosynthetic and ROS on exposure to the waterlogging stress. The decrease in photosynthetic attributes was greater in ‘LA4440’ than that of ‘MIX-002. According to their findings, waterlogging priming boosted the regulation of stomatal conductance, antioxidant enzyme activities, osmotic regulatory chemicals, and critical gene expressions mediated by hydrogen peroxide. However, *Trichoderma harzianum* application did not alleviate the effects of waterlogging stress. The researchers propose that commercially available *Trichoderma harzianum* powder may not function as well as the cultivated strain and/or foliar spraying may not be a good way to apply *Trichoderma harzianum* in a waterlogging stress trial as possible reasons.

The review article by Yang et al. reports that salicylic acid (SA) is known to confer abiotic stress tolerance in horticultural crops and regulates the antioxidant metabolism that scavenges ROS, thereby minimizing oxidative stress in plants. As mentioned in this review, numerous studies have shown that exogenous SA applications in the form of foliar spraying/application to the growing medium play an important role in regulating plant responses to various abiotic stress factors such as salinity, cold, heat, heavy metals, and drought, depending on the plant species. In addition, foliar SA application was found to be beneficial in alleviating the detrimental impacts of water deprivation on plants. SA functions as a signalling molecule in plants, regulating physiological activities such as photosynthesis and growth. SA activates protein kinases that phosphorylate and activate transcription factors involved in stress responses in plants. SA regulates physiological activities in plants such as antioxidant defense system, photosynthesis, carbohydrate metabolism, balanced redox homeostasis, and finally better growth.

Hirakawa et al. reported that NAG is used for chemical priming that alleviates oxidative stress in *Arabidopsis thaliana* via the upregulation of oxidative stress-responsive genes with an increment in histone acetylation levels, and in *Oryza sativa* increased expression of antioxidants enzymes, it acts as priming compound in providing tolerance. The findings imply that NAG, through epigenetic alterations, could improve oxidative stress tolerance and agricultural production in a wide range of plants under environmental stress.

Waheed et al. report on how drought tolerance in Basmati rice genotypes and the identified tolerant lines could be used as potential donors in drought-tolerant rice breeding programs and to identify the genes producing drought tolerance. The identified drought-tolerant lines, on the other hand, should be studied further and recommended as improved Basmati cultivars. **Figure 1** shows a summary of abiotic stress-induced ROS accumulation and mitigation of stress through signalling molecules, discussed in the volume published.

**Figure 1:**
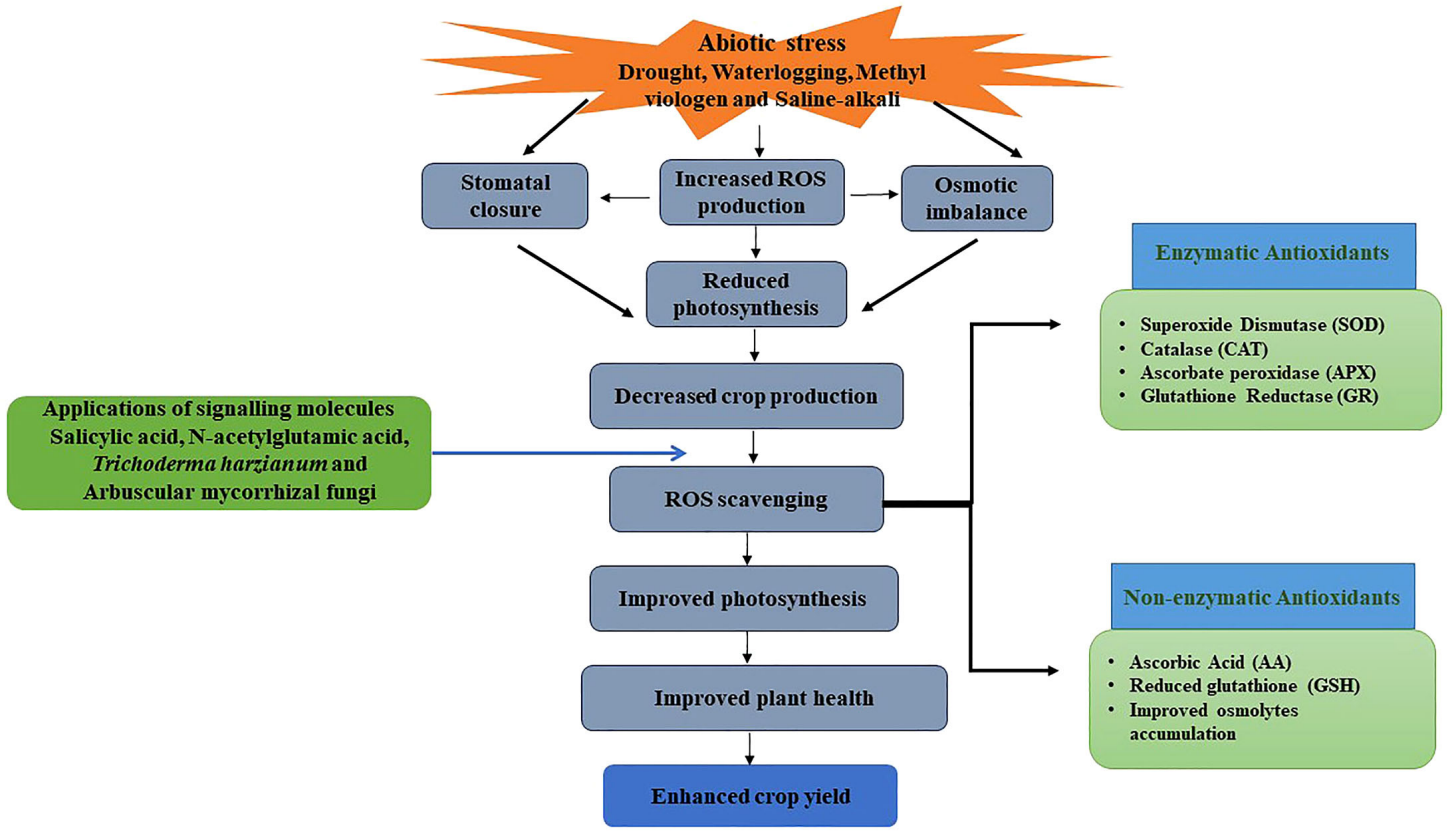
Summary of abiotic stress induced ROS accumulation and mitigation of stress through signaling molecules discussed in the volume published.

These research articles explain how molecular priming contributes to plant abiotic stress tolerance. We anticipate further advances based on our understanding of plant responses, with the goal of establishing new and environmentally benign control mechanisms for abiotic stress tolerance.

## Author contributions

ET: Writing – review & editing. MA: Writing – review & editing.
